# Cytoreductive surgery combined with hyperthermic intraperitoneal chemotherapy and intraoperative radiation therapy in the management of gallbladder cancer: a case report

**DOI:** 10.3389/fonc.2024.1361017

**Published:** 2024-04-03

**Authors:** Marwan Alaswad, Abdullah Al Otry, Ziad Alhosainy, Fadwa Elkordy, Belal Nedal Sabbah, Tarek Ziad Arabi, Humzah Abdulkader, Obaida Fadi Ghanayem, Ayman Zaki Azzam, Tarek Amin

**Affiliations:** ^1^College of Medicine, Alfaisal University, Riyadh, Saudi Arabia; ^2^Department of Surgery, Surgical Oncology, King Faisal Specialist Hospital and Research Center, Riyadh, Saudi Arabia; ^3^Department of General Surgery, Faculty of Medicine, Alexandria University, Alexandria, Egypt

**Keywords:** gallbladder cancer, cytoreductive surgery, hyperthermic intraperitoneal chemotherapy, intraoperative radiation therapy, case series

## Abstract

Gallbladder cancer (GBC) is a rare and highly aggressive malignancy, often characterized by nonspecific clinical presentations and late diagnosis, which contribute to its poor prognosis. It is commonly detected at advanced stages, leading to low survival rates. Surgical resection is the primary treatment, with the extent of surgery depending on the T stage of the cancer. In advanced cases, surgery is only considered if it can potentially be curative. Despite various treatment approaches for advanced GBC, survival outcomes remain poor. In our case series, we introduce a novel treatment approach combining cytoreductive surgery, intraoperative radiation therapy, and hyperthermic intraperitoneal chemotherapy. Remarkably, we observed a 100% one-year survival rate, with one patient achieving eight years of disease-free survival without recurrence or metastasis. This aggressive treatment strategy did not lead to increased morbidity or mortality, suggesting its safety and feasibility. However, larger-scale studies are required to draw definitive conclusions.

## 1 Introduction

Gallbladder cancer (GBC) is a rare, highly aggressive malignancy that constitutes 1.2% of all cancer diagnoses worldwide (1). Additionally, GBC affects 2-3 per 100,000 people and constitutes 1.7% of cancer-related mortality worldwide (1, 2). Several factors have been associated with the development of GBC. Among these, three primary risk factors stand out: having a gallstone larger than 3 centimeters, a family history of GBC, and prolonged cholelithiasis (3, 4). Other risk factors include diabetes, obesity, smoking, gallbladder polyps, and porcelain gallbladder (4–8). Clinically, GBC typically manifests with symptoms that are not unique to this condition, including abdominal pain, fever, jaundice, and weight loss. These symptoms are also common in cholecystitis and other benign gallbladder disorders, which can lead to a delay in diagnosing and treating GBC (9). Moreover, Hawkins et al. concluded that the presence of jaundice at time of diagnosis in patients with GBC is a poor prognostic factor and an indicator of advanced disease, with a median disease specific survival of less than 6 months in GBC patients presenting with jaundice (10). GBC is incidentally discovered in just 1-2% of patients undergoing laparoscopic cholecystectomy, which may contribute to leading its diagnosis at an early stage (11). However, GBC more commonly presents in an ambiguous, delayed manner, which when combined with the anatomic position and vague symptoms, contributes to the advanced stage and the poor prognosis at time of diagnosis (12). Furthermore, only 25% of patients diagnosed with GBC are candidate for surgery at the time of diagnosis, and only 16% of those who undergo surgery achieve an overall survival of more than 5 years (13).

Complete surgical resection remains the only curative option for GBC patients, and the extent of surgical resection is often based on the T stage (14). Recently, aggressive, extensive surgical resection for GBC patients is becoming more prevalent, with longer survival times, and improved overall morbidity and mortality (15–17). In this article, we report five cases of advanced and recurrent GBC treated with a novel approach consisting of cytoreductive surgery (CRS) combined with intraoperative radiation therapy (IORT) and hyperthermic intraperitoneal chemotherapy (HIPEC). A summary of the five presented cases can be found in **Table 1**. Chemotherapy regimens given during the HIPEC phase, along with IORT details can be found summarized in **Table 2**. We followed the preferred reporting of case series (PROCESS) guidelines when reporting our cases (18).

**Table 1:** The clinicopathological characteristics of the five presented cases.

Case	Case 1	Case 2	Case 3	Case 4	Case 5
Age at diagnosis	63	52	54	62	50
Gender	Female	Male	Female	Female	Female
Neoadjuvant chemotherapy	Carboplatin + gemcitabine	None	None	Cisplatin + gemcitabine	Cisplatin + gemcitabine
Tumor type	Adenosquamous carcinoma	Adenocarcinoma	Adenocarcinoma	Adenocarcinoma	Adenocarcinoma
Pathologic TNM	T2N1M0	T2N0M0	T2N2M0	T2N0M0	T2N0M0
Stage	3B	2	4B	2	2
Adjuvant chemotherapy	No	No	Yes, Capecitabine	No	No
Follow up period	1 year	2 years	1 year and 6 months	1 year	8 years
Recurrence	No	No	Yes	Yes	No
Final outcome	Alive	Alive	Dead	Alive	Alive

**Table 2:** Summarized HIPEC and IORT regimens given in our patients.

	Case	Case 1	Case 2	Case 3	Case 4	Case 5
HIPEC	Regimen	Cisplatin + Mitomycin C	Gemcitabine	Gemcitabine	Melphalan	Cisplatin + Mitomycin C
	Dose	Cisplatin 100 mg/m2 Mitomycin C 30 mg/m2	1000 mg/m2	1000 mg/m2	60 mg/m2	Cisplatin 100 mg/m2 Mitomycin C 30 mg/m2
	Circulating time	60 minutes	60 minutes	60 minutes	60 minutes	60 minutes
IORT	Number of applications	2	1	2	1	1
	Site	Porta hepatis and Liver bed	Porta hepatis	Porta hepatis and Liver bed	Porta hepatis	Porta hepatis
	Dose	15 Gray units each	12.5 Gray units	15 Gray units each	12 Gray units	15 Gray units
	Cone size	7 cm each	6.5 cm	7 cm each	9 cm	8 cm
	Beveled angle	30 degrees each	15 degrees	30 degrees each	0 degrees	15 degrees
	Energy used	12 meV each	6 meV	6 meV each	9 meV	6 meV
	Bolus	0.5 cm each	0.5	0.5 cm each	0.5 cm	0.5 cm
	Total depth	2.5 cm each	2 cm	2 cm each	3 cm	2 cm

## 2 Case description

### 2.1 Case 1

A 63-year-old female patient, with a known history of well-controlled hypertension and diabetes mellitus, was referred to our hospital as a case of GBC post-laparoscopic cholecystectomy 5 months prior. Histopathologic examination of the resected specimen revealed an invasive adenosquamous carcinoma of the gallbladder, T2N1M0 stage IIIB.

On admission, computed tomography (CT) scan showed multiple small non-specific hypodense liver lesions at segments 4 and 5, the largest measuring about 0.7 cm, with no signs of local recurrence. Positron emission tomography (PET) and magnetic resonance imaging (MRI) scans revealed findings consistent with the above-mentioned CT results. Complete laboratory workup, along with tumor markers were normal. Alanine aminotransferase (ALT) levels were slightly elevated at 46.7 U/L, aspartate aminotransferase (AST) levels were 26.7 U/L, and alkaline phosphatase levels were 109 U/L.

Based on the imaging findings, we proceeded with six cycles of neoadjuvant chemotherapy with carboplatin and gemcitabine. After completion of the chemotherapy course, the patient underwent CRS in the form of radical cholecystectomy with gallbladder bed resection (segment 5) and upper abdominal lymphadenectomy (aortocaval, retro-pancreatic, and porta hepatis lymph nodes) combined with IORT and HIPEC. Following completion of CRS, we proceeded with IORT and gave two separate applications of 15 Gray (Gy) units each, one at the porta hepatis and one at the liver bed, using a 7 cm cone, at a beveled angle of 30 degrees, 12 Mev energy, with a 0.5 cm bolus, for a total depth of 2.5 cm. After completion of the IORT phase, we proceeded with HIPEC using the open abdomen technique administering cisplatin 100 mg/m2 with mitomycin C 30 mg/m2, circulating for 60 minutes. The patient tolerated the operation very well, with no immediate intra-operative or post-operative complications.

Postoperative histopathologic examination of the resected specimen revealed metastatic adenosquamous carcinoma in three out of the fourteen removed lymph nodes. On follow-up one year later, the patient is alive and doing well, with no signs of local recurrence or distant metastasis.

### 2.2 Case 2

A 52-year-old male patient, known case of GBC who had previously underwent surgical treatment in the form of CRS and IORT. Histopathologic examination of the resected specimen at that time revealed a moderately differentiated adenocarcinoma of the gallbladder, T2N0M0 stage II.

Five years postoperatively, MRI and PET scans revealed an enlarged retrocaval lymph node suggestive of recurrence, with no signs of local recurrence or distant metastasis. This was treated with paracaval lymph node dissection and IORT at the porta hepatis area. Subsequent histopathologic examination revealed metastatic adenocarcinoma in two out of three resected lymph nodes.

Eighteen months after lymph node dissection, a PET scan revealed an FDG-avid enlarged lymph node located posterior to the transverse colon, highly suspicious of recurrence. Due to the location of the lymph node, biopsy of the lesion was not feasible. MRI and magnetic resonance cholangiopancreatography (MRCP) revealed findings consistent with the above-mentioned imaging results. ALT levels were 28.9 U/L, AST levels were 31.1 U/L, and alkaline phosphatase levels were slightly elevated at 122 U/L. All tumor markers levels were normal apart from CEA levels, which were elevated at 22.8 ug/L.

Subsequently, the patient underwent CRS in the form of lymph node resection combined with IORT (at the para-aortic region) and HIPEC. Following completion of CRS, we proceeded with IORT given at the porta hepatis and gave one application of 12.5 Gy units using a 6.5 cm cone, at a beveled angle of 15 degrees, 6 Mev energy, with a 0.5 cm bolus, for a total depth of 2 cm. Then, we proceeded with HIPEC using the open abdomen technique and gemcitabine 1000 mg/m^2^, circulating for 60 minutes. The patient tolerated the operation very well, with no immediate intra-operative or post-operative complications. Postoperative histopathologic examination of the resected lymph nodes revealed metastatic adenocarcinoma in one out of the sixteen removed lymph nodes. Two years postoperatively, the patient is alive and doing well without signs of recurrence or metastasis.

### 2.3 Case 3

A 54-year-old female patient with well-controlled hypertension was referred to our hospital as a case of GBC post-laparoscopic cholecystectomy, including porta-hepatis and retropancreatic lymphadenectomy. Postoperative histopathologic examination of the resected specimen revealed a moderately differentiated adenocarcinoma, T2N2M0 stage IVB. Three weeks later, she presented to the emergency department with epigastric abdominal pain that was associated with vomiting and anorexia. The patient does not have family history of any cancers. On physical examination, the patient looked well and not in distress, with normal vitals. Abdominal examination was insignificant for any tenderness or palpable masses. The rest of her examination was normal.

Laboratory work-up was unremarkable except for an elevated CA-125 level of 38.6 U/mL. ALT levels were 33.6 U/L, AST levels were 21.5 U/L, and alkaline phosphatase levels were 90 U/L. Ultrasound examination revealed chronic portal vein thrombosis with the presence of collaterals. CT scan revealed post-operative fluid collection at the gallbladder fossa, for which residual disease could not be completely excluded, along with partial thrombosis of the left portal vein. PET scan revealed findings consistent with the CT findings, along with no signs of FDG-avid disease or distant metastasis elsewhere. MRI and MRCP revealed findings consistent with the above-mentioned imaging results.

Subsequently, the patient underwent cytoreductive surgery in the form of radical cholecystectomy and gallbladder bed resection (segment 5) combined with IORT and HIPEC, along with Roux-en-Y biliary-enteric anastomosis, appendectomy, and omentectomy. Following completion of the CRS, we proceeded with IORT and gave 2 separate applications of 15 Gy units each, one at the porta hepatis and one at the liver bed, using a 7 cm cone, at a beveled angle of 30 degrees, 6 Mev energy, with a 0.5 cm bolus, for a total depth of 2 cm. After completion of the IORT phase, we proceeded with HIPEC using the open abdomen technique and gemcitabine 1000mg/m^2^, circulating for 60 minutes. The patient tolerated the operation very well, with no immediate intra-operative or post-operative complications. After the operation, the patient was put on adjuvant chemotherapy with capecitabine and completed 8 cycles.

On follow-up six months after the operation, CT scan revealed the development of a new focal hepatic lesion in the left lobe of the liver, along with multiple new, bilateral pulmonary nodules, all indicative of metastasis. This was confirmed by a PET scan. Multiple chemotherapy regimens, including four cycles of gemcitabine and cisplatin, as well as four cycles of FOLFIRINOX (fluorouracil, folinic acid, irinotecan, and oxaliplatin), were tried; however, the patient`s disease was progressive and showed no response to any treatment. Unfortunately, one and a half years post-operatively, the patient passed away due to extensive disease progression.

### 2.4 Case 4

A 62-year-old female patient, known case of hypertension that is well controlled on medications, was referred to our hospital as a case of GBC that was diagnosed incidentally 8 months ago during laparoscopic cholecystectomy. Histopathologic examination of the resected specimen revealed moderately differentiated invasive adenocarcinoma, with positive resection margins, T2N0M0 stage II. The patient denies any family history of cancer. Complete physical examination, along with vitals, were both normal.

Laboratory work-up was unremarkable except for elevated CA 19-9 and CA-125 levels at 61 and 45, respectively. ALT levels were 10.7 U/L, AST levels were 16.3 U/L, and alkaline phosphatase levels were 56.6 U/L. CT scan of the abdomen showed multiple liver hypodensities in liver segments 4A, 4B, and 5, along with an enlarged portocaval lymph node measuring 0.7 mm. PET scan and MRI revealed similar findings. Based on these findings, the patient subsequently received six cycles of neoadjuvant chemotherapy in the form of combined cisplatin and gemcitabine. After completing the neoadjuvant chemotherapy course, the patient underwent cytoreductive surgery in the form of radical cholecystectomy and gallbladder bed resection (segment 5), along with liver metastecotmy, radical lymph node dissection for the area of the porta hepatis, omentectomy, and partial peritonectomy, combined with IORT and HIPEC. One application of 12 Gy units was given at the porta hepatis area, using a 9 cm cone, 0-degree beveled angle, 9 Mev energy, 0.5 cm bolus, for a total depth of 3 cm. After that, HIPEC was performed using the open abdomen technique and 60 mg/m^2^ melphalan (given instead of cisplatin because of known allergy), circulating for 60 minutes.

The patient tolerated the operation very well, with no immediate intra-operative or post-operative complications. Post-operative histologic examination of the resected specimens was positive for metastatic adenocarcinoma in the liver resections and in one porta hepatis lymph node. The patient was offered adjuvant chemotherapy due to the aggressive nature of her disease and the hepatic involvement; however, the patient declined and did not want to take any further chemotherapy. On follow-up examination one year later, the patient is still alive, but CT scan showed development of large hepatic lesions in the left lateral lobe, along with a large mass lesion that cannot be dissociated from the head of pancreas which invades the 2^nd^ part duodenum, and a new mass in the peritoneal cavity, consistent with metastasis and disease progression.

### 2.5 Case 5

A 50-year-old female patient with no prior medical history presented to our emergency department complaining of right upper quadrant abdominal pain, associated with nausea and vomiting. Physical examination, along with vitals were unremarkable except for localized abdominal pain, with no associated rebound tenderness or guarding. Laboratory workup including tumor markers were all normal.

Chest and abdominal X-rays were done and were negative for any abnormal findings. CT scan revealed a mass lesion located at the neck of the gallbladder, which is causing obstruction and gross distention of the body and fundus of the gallbladder. The gallbladder was adherent to the liver, however; no focal intrahepatic lesions were seen, along with no signs of nodal or distant metastasis. A subsequent PET scan revealed an FDG-avid mass located at the gallbladder neck with focal infiltration of adjacent peritoneal fat, consistent with the diagnosis of locally advanced gallbladder cancer. ALT levels were 12.1 U/L, AST levels were 15.1 U/L, and alkaline phosphatase levels were 73.4 U/L; all normal.

Due to the extent of her disease, we proceeded with six cycles of neoadjuvant gemcitabine and cisplatin. Following the completion of her chemotherapy course, CT scan of the abdomen showed good response of the tumor to the neoadjuvant chemotherapy, with no signs of nodal or distant metastasis. Subsequently, the patient underwent CRS in the form of radical cholecystectomy with gallbladder bed resection (segment 5), appendectomy, omentectomy, splenectomy, IORT, and HIPEC. One application of 15 Gy units was given at the porta hepatis area, using a 8 cm cone, 15 degree beveled angle, 6 Mev energy, 0.5 cm bolus, for a total depth of 2 cm. After that, HIPEC was performed using the open abdomen technique and 100 mg/m2 cisplatin combined with 30 mg/m2 mitomycin C, circulating for 60 minutes. The patient tolerated the operation very well, with no immediate intra-operative or post-operative complications. Postoperative histopathologic examination revealed a moderately differentiated, invasive adenocarcinoma of the gallbladder, T2N0M0 stage II. The patient has been following up regularly for the last eight years and is doing extremely well with no signs of recurrence or distant metastasis.

## 3 Discussion

GBC prognosis remains poor due to several reasons, with the most important being the vague, non-specific clinical appearance which often leads to delayed diagnosis (9). Initial ultrasonographic (US) examination is helpful in patients presenting with jaundice, and if US reveals features suggestive of GBC, CT, MRI, and positron emission tomography can be helpful in evaluating the extent of local and distant spread of the disease (19, 20). Furthermore, MRCP has been proven to be a reliable method in assessing local and regional spread of GBC (21).

Traditionally, GBC patients have been enrolled in clinical trials along with biliary tract cancer, hence the regimens of systemic chemotherapy for GBC patients is the same as that of cholangiocarcinoma, with combined gemcitabine and cisplatin regimen being first-line treatment (22). Valle et al. conducted a phase III randomized controlled trial on 410 patients with biliary tract cancer, of which 149 had GBC, and concluded that the addition of cisplatin to gemcitabine was superior to gemcitabine alone in terms of progression free survival (8.4 months vs. 6.5 months) and overall survival (11.7 vs. 8.3 months) (23). Furthermore, the pursuit of alternative therapeutic strategies extends beyond traditional chemotherapy, exploring treatments for particularly challenging cases, including unresectable recurrent rectal cancers and advanced melanomas, reflecting the ongoing efforts to enhance outcomes for difficult-to-treat malignancies (24, 25).

Complete surgical resection remains the only curative option for GBC patients, and the extent of surgical resection is often based on the T stage (14). Simple cholecystectomy has been found to be adequate for the management of T1a GBC, with numerous studies reporting an overall survival rate of 100% (26–28). On the other hand, it is highly recommended that T1b GBC patients should be treated with radical resection involving regional lymphadenectomy (29, 30), with a Markov decision model revealing significantly longer survival period in T1b GBC patients treated with radical resection compared to simple resection (9.85 vs. 6.42 years) (31). Furthermore, radical cholecystectomy with en-bloc resection of the of the liver bed (Couinaud segments 4B and 5) is the preferred treatment for T2 GBC, with an overall 5-year survival rate of 90%, versus 40% for cholecystectomy alone (32). Surgical resection for T3 and T4 GBC should only be done if R0 resection can be achieved, and often includes extensive radical resection in the form of common bile duct resection, right hemihepatectomy, and pancreaticoduodenectomy (33, 34).

CRS and HIPEC has been proven to be effective in prolonging the survival of patients with colorectal adenocarcinoma, gastric adenocarcinoma, appendiceal adenocarcinoma, pseudomyxoma peritonei and peritoneal mesothelioma (35). However, few data are available on the role of this CRS combined with HIPEC in GBC. Randle et al. evaluated the role of CRS combined with HIPEC in 5 patients with GBC and concurrent peritoneal carcinomatosis, and reported a 30% 3-year survival rate, with a median survival of 22.4 months (36). Those findings were supported by Lui et al, who reported a longer median overall survival of 19.3 months in the CRS and HIPEC group, compared to 15.3 months in the control group. They also reported a statistically significant longer hospital stay in the CRS and HIPEC group, however; the rate of post-operative complications was not increased by HIPEC (37). Furthermore, these findings are supported by Amblard et al. who reported a statistically significant longer median overall survival in patients treated with CRS and HIPEC compared to those treated with systemic chemotherapy alone (21.4 vs. 9.3 months) (38).

Due to the rarity of this malignancy, few data regarding the role of IORT in GBC is available, and a consensus on the role of IORT is yet to be reached. Todoroki et al. compared the efficacy of combined CRS and IORT versus CRS alone in 27 patients with stage IV GBC and reported a 3-year cumulative survival rate of 10.1% in the CRS and IORT group, versus 0% for the CRS alone group (39).

CRS combined with IORT and HIPEC has been tried before in rectal cancer (40, 41), peritoneal sarcomatosis (42), and in pancreatic cancer (43), with early but promising results. In this case series, we present five cases of GBC treated with CRS combined with HIPEC and IORT, with an overall one-year survival of 100%. Moreover, one patient has achieved an eight-year disease free survival following treatment. Regarding our treatment regimen choice, our institution initially used the cisplatin + mitomycin C regimen for all hepatopancreatobiliary malignancies, as demonstrated in two patients in this case series. However, we later transitioned to gemcitabine as the gold-standard chemotherapeutic agent for HIPEC in treating all hepatopancreatobiliary malignancies at our institution. This change was based on the institutionally approved guidelines for chemotherapeutic agents used in HIPEC and CRS. To the best of our knowledge, this is the first study that reports the use of CRS combined with HIPEC and IORT in the management of GBC. This technique appears to be successful in achieving local and regional control among GBC patients. Nevertheless, further studies and clinical trials are needed to determine the true efficacy and side effects of this treatment modality in this patient group.

## Data availability statement

The original contributions presented in the study are included in the article/supplementary material. Further inquiries can be directed to the corresponding author.

## Ethics statement

Ethical approval was not required for the studies involving humans because case series do not require an ethical approval at our institution. The studies were conducted in accordance with the local legislation and institutional requirements. The participants provided their written informed consent to participate in this study. Written informed consent was obtained from the individual(s) for the publication of any potentially identifiable images or data included in this article. Written informed consent was obtained from the participant/patient(s) for the publication of this case report.

## Author contributions

MA: Writing – review & editing, Writing – original draft, Data curation, Conceptualization. AbA: Writing – original draft, Data curation. ZA: Writing – original draft, Data curation. FE: Writing – original draft. BS: Writing – original draft. TAr: Writing – original draft. HA: Writing – original draft. OG: Writing – original draft. AyA: Writing – review & editing. TAm: Writing – review & editing.
